# Erectile Dysfunction and Hypertension: Impact on Cardiovascular Risk and Treatment

**DOI:** 10.1155/2012/627278

**Published:** 2012-05-09

**Authors:** Valter Javaroni, Mario Fritsch Neves

**Affiliations:** ^1^Department of Clinical Medicine, State University of Rio de Janeiro, 20551030 Rio de Janeiro, RJ, Brazil; ^2^Departamento de Clínica Médica, Hospital Universitário Pedro Ernesto, Rua Vinte e Oito de Setembro, 77 sala 329, Vila Isabel, 20551030 Rio de Janeiro, RJ, Brazil

## Abstract

Erectile dysfunction (ED) is a common complaint in hypertensive men and can represent a systemic vascular disease, an adverse effect of antihypertensive medication or a frequent concern that may impair drug compliance. ED has been considered an early marker of cardiovascular disease. The connection between both conditions seems to be located in the endothelium, which may become unable to generate the necessary dilatation in penile vascular bed in response to sexual excitement, producing persistent impairment in erection. On the other hand, the real influence of antihypertensive drugs in erectile function still deserves discussion. Therefore, regardless of ED mechanism in hypertension, early diagnosis and correct approach of sexual life represent an important step of cardiovascular evaluation which certainly contributes for a better choice of hypertension treatment, preventing some complications and restoring the quality of life.

## 1. Introduction

Erectile dysfunction (ED) has been defined according to National Institute of Health from 1993 as the persistent inability to reach or maintain and penile rigidity enough for sexual satisfaction [1]. ED has a high prevalence around the world and a huge impact on quality of life of men and their partners [2]. With the increment of life expectation and aging of population, ED burden is supposed to increase in the upcoming years [3].

Common risk factor categories associated with sexual dysfunction exist for men and women including individual general health status, diabetes mellitus, cardiovascular disease, other genitourinary disease, psychiatric/psychological disorders, other chronic disease, and sociodemographic conditions. Actually, vasculogenic ED is considered part of a systemic vasculopathy and has a known relation with cardiovascular risk factors such as hypertension, diabetes, dyslipidemia, and smoking. ED has been considered an early marker of cardiovascular risk that could precede traditional clinical manifestations of atherosclerosis, indicating the presence of vascular disease. In addition, ED could alert clinicians to the presence of unknown risk factors and an increased cardiovascular risk. Thus, ED could offer the opportunity to implement adequate therapeutic efforts to minimize the burden of major cardiovascular disease such as myocardium infarction and stroke [4].

As ED is highly prevalent and deeply impacts overall health of sexually active men, sexual function should be part of anamneses in all hypertensive subjects, especially those over 50 years. Ideally, such investigation could be held before starting therapeutic. To stimulate this attitude, the main objectives of this paper are to review some aspects linking ED and hypertension, including arterial hypertension as a risk factor for ED, ED as a marker of cardiovascular risk, ED and antihypertensive drugs, its possible negative impact in therapeutic adhesion, and lastly, actual therapeutic approach of hypertensive men with ED.

## 2. ED Prevalence and Impact around the World

ED prevalence was surprisingly high at the end of the classic Massachusetts Male Aging Study (MMAS) reaching almost 40% among men at theirs 6th decade of life. Those numbers were similar in more recent publications over the world [5] and also in developing countries [6], projecting the assumption that over 30 million American citizens suffer from some level of sexual dysfunction [5].

Prevalence's numbers vary according to characteristics of the population studied and the method used to access erectile function. Some trials have used a single question about sexual satisfaction while others have adopted validated questionnaires like International Index of Erectile Function (IIEF) that could check all five major domains of sexuality: sexual desire, erectile function, orgasmic function, intercourse satisfaction, and overall satisfaction with sexual life [3].

Besides this high prevalence, before oral therapy with phosphodiesterase-5 (PDE5) inhibitors, less than 1/4 of men with ED search for medical help [7]. The most frequent reasons for such passiveness were belief that lack of complete erection was part of a normal aging, sexual inactivity caused by widowhood, lack of perception of ED as a medical disorder, ashamed to talk with a doctor about sexuality, lack of an effective treatment for most cases. Probably the real impact of ED was even greater with a strong relationship with aging and some authors estimated that almost half of 70-year-old men live with some degree of ED [5, 6].

After introduction of sildenafil in therapeutic market in 1998 [8], a revolution shook this scenario and search for offices increased as well as medical knowledge about ED and the way physicians treat their patients [9]. From the label of having an psychological illness to an exhaustive, invasive and mostly usefulness series of complementary exams, the evaluation post-PDE5 inhibitors turned to a simple identification of risk factors [10], their control whenever possible, and improvement of sexual performance through PDE5 inhibitors prescriptions [11] that quickly became one sales blockbuster.

As a consequence of PDE5 inhibitors basic development studies, erectile process was better understood and several papers from the last decade stressed the association between ED and vascular disease identified by functional and structural changes related to atherosclerosis process [12]. These evidences, in addition to the mechanism of action of such drugs—based on dilation of muscular layers of arteries and cavernous spaces by the blockage of cyclic GMP degradation—point out ED as part of a generalized vasculopathy [13].

It seems important to remember the complexity of erection physiopathology as well as of the hypothetical link with cardiovascular disease—endothelial dysfunction—since multiple factors could cause ED and interfere in the delicate balance of mediators released from endothelium [14]. But psychological aspects of man sexuality interfere in all steps of sexual disorders and could complicate diagnostic attempts or harm therapy efforts. It is really important to individualize each complain in order to understand the situation and offer the best medical approach.

New therapeutic strategies and molecular targets will help to improve quality of erections and sexual satisfaction. In order to cure ED, if it is really possible, some recent studies propose regular use of drugs with proved endothelial action such as statins or PDE 5 inhibitors, taken daily instead of on demand [15], in order to provide sensation of been always ready for intercourse. Medical advances apart, the best treatment for ED remain its prevention. In this sense, men knowledge about cardiovascular health and the relationship of ED with traditional risk factors should help physicians to motivate therapeutic adhesion and adoption of a healthier way of life [16].

## 3. ED and Cardiovascular Risk

Several traditional risk factors were related to ED in medical literature with some evidences coming from well designed epidemiological trials. Age seems to be the clearest risk factor with strong association with the presence and severity of ED [5]. After adjusting for age, the correlation between ED and modifiable risk factors—hypertension, diabetes, hyperlipidemia, obesity, sedentary, and smoking—remained significant [17]. The increase of ED's prevalence with aging is followed by atherosclerotic lesions in vascular tree [18]. Most men with hypothetic vasculogenic ED present at least one traditional cardiovascular risk factor [19]. These evidences allowed the consideration of ED as a clinical manifestation of a functional (lack of vasodilation) or structural abnormality in penile circulation as component of a systemic vasculopathy [20].

Erection is a complex psiconeurovascular process and involves several system interactions that converge to an increase in hypogastrian-penian blood flow and subsequent activation of veno-occlusive mechanism of corpus cavernosum [21]. It is well known that the blood increment towards cavernous tissues necessary for a rigid erection is huge and even small hemodynamic disturbances could produce sexual dysfunction [22]. So, traditional risk factors such as hypertension, diabetes, and hyperlipidemia could contribute for ED development or worsening even in situations where psychological etiology seems more likely.

In addition, penile erectile tissue's integrity depends on oxygen tension fluctuations that occur during physiologic erections. As a consequence of regular erections, several cytokines, vasoactive, and growth factors keep a suitable environment for erectile tissue with a protective effect over stroma and muscular cells of this region [23]. In a pathological condition causing the absence of stimulated or physiological erections and abolishing such stimulus, there would be a structural change in tissue composition with deleterious consequences on erectile capacity [24].

Some trials have shown the presence of vascular disease in men suffering from vasculogenic ED but without traditional risk factors, pointing out ED as a clinical early cardiovascular risk marker [25]. Particularly among men less than 60 years old, ED seems to act as a risk factor independent of traditional markers [26]. More recently, laboratorial markers such as dimetilarginin asymmetric (ADMA) [27] and C-reactive protein were reported higher in ED men when compared with men without ED and similar risk factors [28].

On the other hand, as a consequence of its multifactorial aspect, several conditions could promote ED without systemic vascular involvement such as pelvic surgeries, depression, Peyronie's disease, and prostatism. Probably this aspect is one among others to explain the lack of additional contribution of ED over traditional risk factors (Framingham score) during cardiovascular evaluation in some reports [29]. Other possible explanations were the characteristics of study population, method of assessing risk factors and the diagnostic tool used for ED diagnostic.

Other risk factors have been related to ED. Sedentarism, obesity, and smoking have been implicated in the etiology of ED, and an approach of these risk factors has been able to reverse ED and restore normal erectile function [30]. Metabolic syndrome and waist-to-rip ratio have been associated to more severe ED among those over 50 years old [31]. Sleep disorders were also more prevalent among ED men and their treatment could help in recovering sexual satisfaction [32].

## 4. Erectile Dysfunction and Systemic Hypertension

Hypertension is considered one of the most hazardous cardiovascular risk factors and it is a frequent comorbidity of men with ED [33]. One of the first studies to ask about sexual function among hypertensives was the classic TOMHS (*The Treatment of Mild Hypertension Study*) [34] and its results contributed to the false belief that ED was rare in this population since they found only 12.2% of men referring any degree of sexual dysfunction at inclusion. TOMHS excluded subjects with comorbidities like diabetes or hyperlipidemia, older and moderate or severe hypertension. At the end of TOMHS, ED was more frequent among those with more antihypertensive drugs or systolic pressure over 140 mm Hg. Other trials also refuse the high prevalence of ED among hypertensives [35] probably due to characteristics of the sample and the method to diagnose ED.

On the other hand, Jensen et al. observed that the main reason for ED among hypertensive individuals were penile circulation disability (found in 89%), probably due to atherosclerosis [36]. Burchardt and coworkers using IIEF-5 to access erectile function among hypertensive men, aging from 34 to 75 years old, found 68.3% of ED prevalence [33]. Feldman et al. stressed the significant association between ED and traditional risk factors for coronary artery disease, like hypertension, smoking, overweight and hyperlipidemia [5]. Giuliano et al. performed a survey of 7689 patients (mean age 59 years) using the Sexual Health Inventory in Men (SHIM) questionnaire. In 3906 men with hypertension alone (no diabetes), ED was present in 67% (defined as a SHIM score of 21) [37]. Doumas et al. studied Greek hypertensive patients and identified ED in 35.2%, which correlated with age, duration of hypertension and use of antihypertensive drugs [38]. Mittawae et al. evaluated the sexual function of 800 Egyptian hypertensive subjects and found ED in 43.2% and duration of hypertension was the variable with stronger correlation with ED severity [39]. Recently, Chang et al. suggested that ED severity was associated with number of risk factors, including criteria for metabolic syndrome [40]. A retrospective analysis of placebo group of Prostate Cancer Prevention Trial estimated the 5-year risk of coronary events among men with ED in 11% which, in terms of preventive medicine, means that ED could be considered an equivalent of coronary disease [41].

## 5. Erectile Dysfunction and Antihypertensive Drugs

The association of ED and vascular risk factors including hypertension raises the hypothesis that endothelial dysfunction is the common link between erectile dysfunction and cardiovascular disease. But a possible association between ED and hypertension is much more intricate issue involving other aspects, such as the hemodynamic interferences caused by antihypertensive drugs.

**Figure 1:**
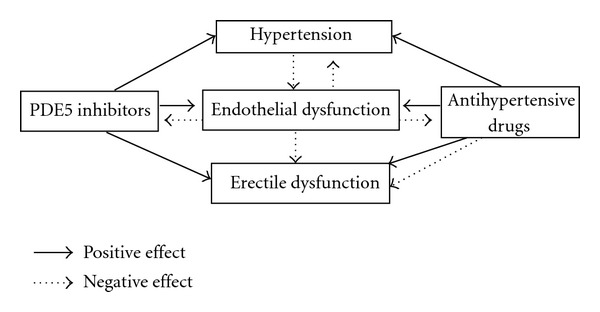
Relationship between hypertension and erectile dysfunction. PDE5, phosphodiesterase 5.

There is a complex relationship among arterial hypertension and erectile dysfunction that is explained by the multifactorial pathophysiological process that take place in both conditions (Figure 1). So, considering that it is still matter of discussion if hypertension is cause or consequence of endothelial dysfunction, it can influence ED severity or it could appear before ED. Depending on the class of the antihypertensive drug and its effect over endothelium mediators, the impact on ED could be positive or negative. PDE5 inhibitors, ED's first line therapy, present a mechanism of action based on NO bioavailability. Lack of efficacy could represent a more intense vascular damage. In contrast, continuous use of PDE5 inhibitors proved to reverse endothelial dysfunction with positive impact on sexual function and even on blood pressure control. 

In this sense, it is an usual popular belief to blame medical therapy for hypertension as the main reason of erectile problems, especially when there is a temporal coincidence of starting symptoms and use of antihypertensive drugs, in particular when including the “old” diuretics and beta-blockers [34]. In almost all trials where this topic was studied, ED was not the primary objective and was assessed by patient reports instead of questionnaire evaluation or measurement of penile rigidity. So, there is a lack of definitive evidence even with betablocker and diuretics. Recently, Baumhäkel et al. performed a systematic analysis of trials evaluating erectile function with a validated questionnaire or direct assessment of penile rigidity and concluded that only thiazide diuretics and betablockers, not including nebivolol, may influence erectile function. ACE inhibitors, angiotensin receptor blockers, and calcium channel antagonists were reported to have no relevant or even a positive effect on erectile function [42]. 

Development of erectile dysfunction in connection with betablockers might be biased by psychological effects derived from the awareness of being treated with a certain substance. This is an important point since patient concerns about the adverse effects of drugs on erectile function might limit the use of essential medications in cardiovascular high-risk patients [43].

In the same way, data with diuretics and ED are not conclusive. A small number of patients and inadequate evaluation of erectile function limit the trials results. On the other hand, the erectile dysfunction substudy in ONgoing Telmisartan Alone and in combination with Ramipril Global Endpoint Trial/Telmisartan Randomized AssessmeNt Study in ACE iNtolerant subjects with cardiovascular Disease (ONTARGET/TRANSCEND) did not demonstrate a significant association of pretreatment with diuretics with erectile function in high-risk patients [44].

It is important to consider that drugs used for treatment of cardiovascular diseases have often been accused of influencing erectile function, and such belief could influence drug compliance [42]. Some authors do not agree with a class specific and constant effect over erectile function [45]. Others believe that the hypotensive effect of any drug could produce ED in susceptible subjects with comorbidities [36]. More recently, Earden et al. showed that the presence of “*nondipper”* pattern on ambulatory blood pressure monitoring among treated hypertensive patients were strongly related to worst erectile function independently of the number or the class of antihypertensive drugs in use [46]. On the other hand, small studies suggested that some antihypertensive drug classes could have less harmful or even beneficial effect on sexual function like calcium channel antagonists [47], angiotensin II receptor blockers [44], and nebivolol [45].

There is no clinical trial evaluating the effect of calcium channel antagonists on erectile function with an adequate assessment of ED, but they are reported to have no relevant effect on erectile function [47]. For drugs that act over renin-angiotensin system, most evidence suggests that there was no influence on erectile function, and some authors indicate beneficial effect [42]. However, in 1549 cardiovascular high-risk patients included in ONTARGET/TRANSCEND trial, there was neither a beneficial effect of the ACE inhibitor ramipril, the angiotensin receptor blocker telmisartan, nor of the combination of both on erectile function [48].

Favorable effects on nitric oxide synthase and oxidative stress have been shown with nebivolol pointing out a mechanism for improvement of erectile function. Experimental studies have demonstrated an enhancement of endothelial function in aorta and corpus cavernosum with a significant reduction in penile oxidative stress and collagen content [49], protected cavernosal tissue against structural changes, and increased expression of endothelial NO synthase (eNOS) [50].

Although no class of antihypertensive agents presents a clearly superior effect over the others in terms of quality of life, the current impression is that nebivolol, ACE inhibitors, and angiotensin II receptor antagonists may offer some advantage, at least in regard to effects on cognitive function and sexual activity [51].

The presence of comorbidities and concomitant drugs, a common situation in older hypertensive subjects, and lack of diagnostic standardization concerning tools to access erectile function impair a reliable analysis of trials about the relationship between ED and hypertension as well as any robust conclusion about deleterious action of antihypertensive drugs on erectile function [52]. In this way, search for new data on basic mechanism under ED development in hypertensive individuals is an actual need. In an individual aspect, sexual activity and erectile function quality should be part of anamnesis before starting antihypertensive therapy and seems to play a relevant role in the followup, as it would allow a scalable monitoring of erectile function, help the selection of better classes of antihypertensive drugs, turn easier the identification of adverse sexual events, and even improve therapeutic compliance [51].

## 6. Pathophysiology of ED in Hypertensive Patients

Several hypotheses try to explain the pathophysiology of ED in hypertensive individuals. Since the pioneering work of Jeremy et al. [53] who highlighted the role of endothelial oxidative stress on the genesis of ED, other publications reinforce this finding [54] even demonstrating that antioxidant therapy could improve endothelial function with positive interference on erectile function [55].

Old and more recent experimental studies have pointed out the role of nitric oxide (NO) and other possible mediators in endothelial dysfunction of hypertensive rats [56]. In spontaneously hypertensive rats, endothelial-mediated relaxation of corporal cavernosal strips in response to acetylcholine was significantly impaired, suggesting a defect in endothelium-dependent reactivity and a corresponding reduction in NO [57]. Perticone et al. demonstrated that endothelial function of hypertensive patients had an inverse relationship with L-arginin (NO precursor) levels and ADMA, a competitive inhibitor of eNOS [58]. On the other hand, decrease in NO production or bioavailability would take place in the etiology of hypertension in several clinical situations [59] where ED is not always present. Thus, one possible mechanism by which hypertension may cause ED is likely related to endothelial dysfunction associated with hypertension. Long-standing hypertension may cause oxidative stress, endothelial cell injury, and its consequences, including the inability of arteries, arterioles, and sinusoids of the corpus cavernosum to dilate properly [60]. Some authors state that ED symptoms in hypertensive patients would represent deterioration in endothelial dysfunction already present and should alert for a possible progression of a systemic vasculopathy [61].

Corroborating this link among hypertension and ED, Vlachopoulos et al. identified that hypertensive men had greater carotid intima-media thickness, lower brachial flow-mediated dilation, and higher levels of serum inflammatory mediators than normotensive individuals with similar cardiovascular risk [62]. They suggested NO bioavailability reduction caused by ADMA accumulation consequent of high blood pressure as the molecular mechanism for these findings. This and other authors [63] argue that ED should represent a clinical sign of a deeper vascular damage in hypertensive patients and an increased risk of cardiovascular events. In this way, Prisant et al. showed that ED severity among hypertensive subjects were associated with age, duration of hypertension, and presence of peripheral vascular disease [64].

During COBRA (PolarCath Cryoplasty Versus Conventional Balloon Postdilation of Nitinol Stents for Peripheral Vascular Interventions) trial, Montorsi et al. evaluated 285 subjects with proved coronary arterial disease and identified that ED symptoms began 2 to 3 years before clinical manifestation of coronary disease [61]. Robust clinical trials confirm that ED is a strong cardiovascular event predictor such as cardiovascular death, myocardial infarction, and cardiac failure [48]. Chung et al. followed a cohort of ED men and compared the 5-year encephalic vascular accident risk with another cohort with similar cardiovascular risk but normal erectile function. They confirmed ED as a clinical independent marker of increased cerebral vascular disease risk [65].

As previously commented, the link between both conditions seems to lie in the endothelium which performs many functions that contribute to homeostasis and prevention of atherosclerosis. Endothelial dysfunction is considered one of the earlier steps of atherosclerosis process, preceding angiographic, ultrasonographic, and clinical evidences of vascular disease [66]. These clinical manifestations rarely are symmetrical in the same patient, probably due to different sizes of arterial tree that irrigate territories such as penile, heart, brain, and legs [20], and different needs of blood supply and vasodilation of each structure [67]. It has been suggested that the small cavernosous arteries were more vulnerable to atherosclerosis when compared to larger arterial trees like coronaries [20, 67] explaining why ED precedes angina in a patient with systemic vasculopathy.

The role of NO as a major mediator of erectile pathways such as NO-GPMc and RhoA/Rho-kinase was well documented [68]. Some experimental studies confirmed the participation of eNOS during erectile response [69]. In penile circulation, eNOS activity and NO bioavailability were regulated by several molecular mechanisms such phosphorilation, protein interactions, and oxygen reactive species that control eNOS activity in physiological conditions and could explain several situations where NO bioavailability could decrease and manifest as ED [21].

Clinical evidences seem to confirm this increased vulnerability of cavernosous circulation. Kaiser et al. observed that NO-GMPc pathway was earlier damaged in atherosclerosis process when they followed ED men without cardiovascular disease and compared them with men with normal erectile function. They found that vasodilation of the brachial artery by both mechanisms, endothelium-dependent and independent, was significantly compromised in the group suffering from ED, which illustrates that vascular alterations involved in ED are a generalized process [67]. Accordingly to these authors, possible reasons for the precocity of ED as clinical manifestation of systemic atherosclerosis were the small size of cavernosous arteries and the need of a huge dilation, nearly 80%, in penile circulation for an adequate blood supply for a full erection. This value contrasts with the percentage of other territories where the dilation varies from 10 to 20%. In addition, penile vascular tree seems to be particularly dependent of NO as it participates on arterial dilation to rapidly increase blood flow but also mediates cavernosous sinusoids and venous dilation that represents a crucial step on venoclusive mechanism that assure the obtaining and maintaining of a rigid erection. In several other vascular territories, NO participation on venous side of circulation is minimal or null [67].

Although aging and atherosclerosis are recognized risk factors for ED development, according to some experimental studies the mechanism under those situations is distinct [57]. While ED in atherosclerotic rabbit was a consequence of endothelial and vascular smooth muscle cell dysfunctions in consequence of ADMA accumulation [70], these were not found in older animals with ED but without atherosclerosis where possible pathophysiological mechanism would be the hyperactivity of Rho/RhoKinase pathway [57].

Due to the lack of large clinical trials with long followup period designed for ED identification and with cardiovascular morbidity and mortality as endpoints, it is premature to assure that ED identifies an increased cardiovascular risk among hypertensive patients that extends beyond the risk represented by blood pressure elevation [62]. Studies with larger samples contradict small trials conclusions as they have not observed any association of ED and antihypertensive drugs, cholesterol levels, and even smoking [44]. New randomized trials with appropriate design to clarify these issues are still expected.

## 7. Therapeutic Approach of Hypertensive Patients with ED

 Firstly, it is important to provide an adequate room to talk about sexual dysfunction. It is crucial to listen carefully what patient would like to explain and also to make some questions to check what was understood. The usefulness of standard questionnaires [71] during daily clinical practice is low but the short form of IIEF (5 questions), for example, could help to confirm the ED diagnosis and to provide a clear tool to quantify the pathology and also to follow its evolution and clinical response to therapy [72].

Initial efforts should identify and treat all modifiable risk factors. Next step is a trial with PDE5 inhibitor for those hypertensive subjects without contraindications for sexual activity or for this class of drugs [11]. Thus, knowledge of cardiovascular risk of men with ED is essential, since it could help to identify hidden risk factors and also to evaluate the existence of any contraindication for ED therapy as detailed in the second Princeton consensus [73]. In this way, hypertensive patients would be classified as low, intermediate, or high risk. In case of high risk, before sexual attempts, a cardiologic evaluation is necessary in order to improve conditions to support such metabolic demand [74]. In high risk subjects, cardiologic condition should be stabilized through medication, surgery, or other means before sexual rehabilitation. In intermediate risk, a deeper cardiovascular evaluation is necessary and efforts to improve clinical condition should precede sexual efforts [73].

To assess the individual risk for cardiovascular diseases, repeated measurement of blood pressure and biochemical profile, including fasting serum glucose, creatinine, and lipids, is necessary. Body mass index, according to weight and height, assessment of lifestyle, actual level of physical activity, and potential genetic predisposition should also be obtained. A resting electrocardiogram, should also be documented. If a patient has three or more atherogenic-risk factors, an exercise electrocardiogram should be considered as per the Princeton Consensus Panel. In some high risk patients, a Doppler-sonographic examination of the carotid arteries and lower extremity arteries might also be included into the workup [73].

Cardiovascular disease is not a contraindication to sexual activity in patients who have been properly assessed and treated. In well controlled hypertensive subjects who take one or two antihypertensive drugs and have no other risk factors, indicating low cardiovascular risk, PDE5 inhibitors could be tried. However, among those without treatment or with inadequate blood pressure control or with severe hypertension, it is recommended the cardiologic approach and initial cardiovascular therapy before the prescription of specific drugs to improve erectile function [74].

Patients with diabetes suffering from ED are at special risk for silent cardiovascular disease [75]. Therefore, some authors recommend an exercise test for every diabetic patient presenting with ED, as a significant number of patients with silent ischemic heart disease will be detected [76]. If the results suggest an increased risk for cardiovascular disease, a referral to a cardiologist is reasonable for detailed diagnostic testing and initiation of therapy [73]. Importantly, the heart conditions should be carefully clarified before initiation of medical treatment, as PDE5 inhibitors must not be used in certain cardiovascular conditions or at least require special precaution. In addition, detailed recommendations for cardiovascular patients, concerned about a potential risk of sexual intercourse in the light of their underlying cardiovascular condition, are available both for further diagnostic workup and therapeutic interventions according to the first and second Princeton Consensus Conference [74, 77].

Considering PDE5 inhibitors mechanism of action, it is reasonable to expect for vascular hemodynamic interferences, effects on blood pressure and coronary circulation alterations, especially among hypertensive patients or those on several antihypertensive drugs. However, blood pressure lowering effects of the current PDE5 inhibitors are low, although oral administration of sildenafil was able to reduce systolic and diastolic blood pressure by 7–10 mmHg in a non-dose-dependent manner [78]. When PDE5 inhibitors are administered to patients with hypertension who are taking most antihypertensive agents (e.g., betablockers, angiotensin-converting enzyme inhibitors, angiotensin receptor blockers, calcium antagonists, diuretics), there are usually small additive decreases in blood pressure without a significant increase of adverse events. Similarly, effects of sildenafil on blood pressure in hypertensive patients on multiple antihypertensive drugs were minimal and well tolerated [79, 80]. Comparable results were obtained with newer PDE5 inhibitors vardenafil and tadalafil [80]. Some patients develop orthostatic hypotension when PDE5 inhibitors are used in conjunction with an alphablocker (typically for urologic conditions, such as benign prostatic hypertrophy) but some studies suggest that the interaction is less clinically relevant if the patient has been undergoing long-term alpha-blocker therapy [81]. On the other hand, some beneficial effects on blood pressure control have been shown with PDE5 use in hypertensive patients [82].

Although PDE5 inhibitors have been extensively used for more than one decade, inadequate use is still one major cause of therapeutic failure. Several studies showed that more than a half of ED men leave offices with prescription of PDE5 inhibitor but without correct information about its posology [83]. Most common mistakes include high fat meal near intake, lack of sexual stimulation after intake, short time before sexual intercourse, and low number of attempts [84].

After cardiovascular risk stratification and adequate control of modifiable risk factors, ED patients should be oriented about the right way of sexual medication usage. Although it could sound obvious for doctors, it is prudent to emphasize basic orientation (Table 1) as improper drug usage is a common reason for lack of efficacy.

- Take PDE5 inhibitor at least one hour before sexual intercourse (two hours when using tadalafil).
- Do not take PDE5 inhibitor near (less than two hours) high fat meal or excessive alcohol consumption.
- Sexual stimulation is essential for PDE5 inhibitors action.
- Try more than once and ideally in different situations before giving up.

Even when patients return claiming nonresponse to PDE5 inhibitor, it is recommended to check all steps where medication were tried and review psychological conditions, partner collaboration, blood pressure control, glucose and cholesterol levels [83]. Low total testosterone has been implicated in PDE5 failure. In countries where men have free access to sexual drugs, the situation of improper use is even more dramatic.

When clinical failure with PDE5 inhibitor is confirmed, urologist should participate and evaluate possible options to allow an active and satisfactory sexual life. Intracavernosous self-injection and penile prostheses constitute the most common path for men refractory to oral therapy [11]. But besides referring to specialist, such lack of efficacy should be interpreted properly. We recently showed that hypertensive men with ED who did not respond to 20 mg of vardenafil had lower flow mediated dilation and greater intima media thickness, in addition to a higher Framingham score, pointing to a direct clinical way to identify patients who deserves special attention and probably more aggressive cardiovascular therapy [85].

## 8. Why Asking about Sexual Life before Starting Antihypertensive Drugs?

 Management of hypertension should take into account, especially in elderly, the possible negative impact of antihypertensive drugs on the patient's quality of life, the deterioration of which may reduce treatment compliance [51]. Although ED has a high prevalence in the hypertensive population, sexual questions are not frequently asked during general practitioners consultations [37]. Many hypertensive men do not recognize that they have ED and only a minority of GP considers ED or other sexual issues for the treatment of hypertension as either a possible adverse outcome or as a factor to consider in treatment decision [86].

Knowledge about sexuality seems important as men and women increased their life expectance and want to keep quality of life during aging. Erectile function preservation is essential for sexually active man while having a lower weight for other who has no intercourses. A sexual evaluation in GP visits could detect ED earlier and also help in risk factors therapy customization, avoiding certain drug classes that could disturb sexuality [87]. Besides high prevalence of ED among hypertensive individuals, possible association to ED severity and important aspects in therapeutic compliance improvement justify the relevance of a brief sexual anamnesis in one of the first visits inside GP or cardiologists offices [88]. Evidences demonstrate that the successful treatment of ED is associated with significant improvements in overall physical and emotional well-being [89].

Another interesting aspect of ED knowledge in hypertensive subjects approach is that some evidences point out to an improvement in blood pressure control maybe as a consequence of better therapeutic compliance after PDE5 inhibitor therapy [82]. Likewise, Lamina et al. have shown that hypertensive patients who start better life habits have got an improvement in erectile function simultaneously to serum inflammatory markers reduction, like C-reactive protein [90].

Recent guidelines establish that patients without obvious etiology for ED, such as anatomical disorders, neurological disease, or endocrine causes, should be evaluated for cardiovascular risk factors, arterial hypertension, and arterial disease [91]. The relatively high probability of detecting potentially serious diseases warrants further investigations [75]. In addition, the strong association between cardiovascular risk factors and ED should be brought to the patient's attention, as this, in some patients, might be a more convincing motivation to modify these risk factors, change lifestyle, effectively treat hypertension, quit smoking than more abstract statistical association between cardiovascular disease, and risk factors [92]. ED offers a unique chance to undergo medical examination and, therefore to, improve not only their sexual but, most importantly, their overall health.  Table 2 highlights main reasons to take time and ask about sexual life before starting antihypertensive therapy during next consultation. 

## 9. Conclusions

Despite high prevalence of ED among hypertensive men, it is not often matter of discussion during consultation with general practitioners. An adequate approach to identify sexual health in primary care is needed to identify sexual dysfunctions earlier and also to individualize treatment, to prevent that this situation contributes to a reduction of therapeutic adhesion and even worsens the quality of life among people who already suffer from AH and other traditional risk factors.

Sexual function knowledge by general practitioners and cardiologists is relevant and should be ideally accessed before starting therapy for hypertension. In addition to the high prevalence of ED among hypertensive men, its association with the severity of hypertension, and possible interference after the introduction of antihypertensive medications justify the importance of including a brief sexual anamnesis during clinical consultations.

After ED diagnosis and for those without contraindication for sexual activity or PDE5 inhibitors usage, doctors must take time to adequately inform their patients about the correct way to take sexual medication. Oral therapy failure could represent a deeper vascular damage and worst cardiovascular risk and generally requires the aid of the urologist in order to restore hypertensive man sexual life.

## Figures and Tables

**Table 1:** How to adequately orientate during PDE5 inhibitors prescription.

Make a cardiovascular evaluation that allows you know risk stratification.	
If sexual intercourse is permitted but PDE5 inhibitors not—refer to urologist.	
Use adequate PDE5 inhibitors dosage.	
Inform about the time interval to take drug before sexual intercourse.	
Avoid fat meal or alcohol intake near PDE5 inhibitor consumption. Alert about the necessity of sexual excitement to reach an erection under PDE5 inhibitor.	
Talk about psychological influence on PDE5 inhibitors efficacy-believe it.	
Try at least four to six times in different situations before giving up.	

PDE5, phosphodiesterase-5.

**Table 2:** Reasons to ask about your patient's sexual life during your next consultation.

Increase empathy opportunity to improve doctor and patient relationship.	
High prevalence of ED among hypertensive men. Patients concerns about the adverse effects of drugs on erectile function.	
Meaning of ED as a cardiovascular risk marker.	
Therapeutic options for ED easily available and with good efficacy. Knowledge of PDE5 inhibitor failure and its cardiovascular implication.	
Knowledge of possible sexual adverse effects during antihypertensive treatment.	
Better therapeutic adhesion for drugs and life habits modification.	
Positive impact on patients and their partner quality of life.	

ED, erectile dysfunction; PDE5, phosphodiesterase-5.
